# 

**Published:** 1914-11-07

**Authors:** 


### Medical Appointments.

Mr. Thomas W. Jackson, M.B., Ch.B. Edin., has been appointed to the post of resident surgical officer at the Bradford Royal Infirmary.

Dr. David McKinlay Reid, the fourth assistant medical officer at Horton Mental Hospital, having resigned his position, Mr. N. K. Foster, the fifth assistant, has been promoted to fill the post.

Mr. P. O'C. Finigan has been confirmed by the Metropolitan Asylums Board in his appointment as whole-time dentist to the institutions under the control of the Children's, Asylums, and *Exmouth* Committees.

### Transfer between Bradford and Leeds.

The first batch of wounded soldiers, numbering thirty-nine, reached Bradford on Tuesday, October 27. They came from the 2nd Northern General Hospital, Leeds, which is acting as a distributing centre for the northern part of the West Riding. Twenty cases were admitted to the Royal Infirmary and nineteen to the Royal Eye and Ear Hospital. On Friday last week a further batch of eighteen soldiers arrived and were distributed as follows: thirteen to the Royal Infirmary and five to the Royal Eye and Ear Hospital.

The majority of the first batch were not very serious cases, and twenty-six of them have been passed on to Woodlands Convalescent Home, where also thirty-two wounded Belgian soldiers arrived on Friday.

### Personalia.

Mr. J. A. Dawes, M.P., has been appointed deputy-chairman of the Public Health Committee of the London County Council.

The London County Council have accepted the resignation of Mr. Ralph Browne-Carthew, an assistant medical officer in its public health department.

Mr. F. R. Anderton, Mr. Frank Briant, Sir Shirley Murphy, and Mr. O. E. Warburg have been appointed to represent the London County Council upon the Central Council for London on District Nursing, established as a result of the conferences convened by the Local Government Board.

Mr. Chas. H. Budd, M.B., Ch.B. Oxon., late house-physician at St. Thomas's Hospital, London, and at Addenbrooke's Hospital, Cambridge, who was recently appointed honorary anæsthetist at the latter, has been gazetted captain at the 1st Eastern General Hospital, Cambridge, R.A.M.C.(T.).

### Buildings Contemplated.

Barnet.—The Barnet Board of Guardians invite tenders for the erection of a new infirmary at Welhouse Lane, Barnet. Messrs. Williams and Cox, 34 Henrietta Street, Covent Garden, W.C., are the architects, from whom quantities and forms of tender may be obtained.

Bradford.—The Local Government Board have held an inquiry into an application of the Corporation for sanction to a loan of £100,000 for acquiring the site and buildings of the Bradford Royal Infirmary, and for making a capital contribution towards the provision of new infirmary accommodation by the Board of Management of the Bradford Royal Infirmary.

Ecclesall.—The Ecclesall Bierlow Guardians have resolved to build a children's hospital at a cost of £5,842, exclusive of furnishing.

Foxhall Heath.—The Ipswich Town Council have agreed to build a smallpox hospital at Foxhall Heath. The Public Health Committee have instructed Mr. J. S. Carmenter to get out quantities so that tenders may shortly be invited. The surveyor's total estimate for the building is £5,110.

